# The Zuo Jin Wan Formula Induces Mitochondrial Apoptosis of Cisplatin-Resistant Gastric Cancer Cells via Cofilin-1

**DOI:** 10.1155/2016/8203789

**Published:** 2016-10-30

**Authors:** Qing-Feng Tang, Jian Sun, Hui Yu, Xiao-Jing Shi, Rong Lv, Hong-Chang Wei, Pei-Hao Yin

**Affiliations:** ^1^Central Laboratory and Department of Clinical Laboratory, Putuo Hospital, Shanghai University of Traditional Chinese Medicine, Shanghai 200062, China; ^2^Basic Medical College, Shanghai University of Traditional Chinese Medicine, Shanghai 201203, China; ^3^Department of General Surgery, Putuo Hospital, Shanghai University of Traditional Chinese Medicine, Shanghai 200062, China

## Abstract

Despite the status of cisplatin (DDP) as a classical chemotherapeutic agent in the treatment of cancer, the development of multidrug resistance often leads to a failure of DDP therapy. Here we found that phosphorylated cofilin-1 (p-cofilin-1) was overexpressed in the DDP-resistant human gastric cancer cell lines SGC7901/DDP and BGC823/DDP, relative to the respective parent cell lines (SGC7901 and BGC823), and that DDP induced the dephosphorylation of p-cofilin-1 in both parent lines but not in the DDP-resistant lines. However, we noted that the traditional Chinese medicine formula Zuo Jin Wan (ZJW) could induce the dephosphorylation of p-cofilin-1 and promote cofilin-1 translocation from the cytoplasm into the mitochondria in both SGC7901/DDP and BGC823/DDP cells. This mitochondrial translocation of cofilin-1 was found to induce the conversion of filamentous actin to globular-actin, activate mitochondrial damage and calcium overloading, and induce the mitochondrial apoptosis pathway. We further observed that these effects of ZJW on DDP-resistant human gastric cancer cell lines could be reversed via transfection with cofilin-1-specific siRNA, or treatment with a PP1 and PP2A inhibitor. These results suggest that ZJW is an effective drug therapy for patients with DDP-resistant gastric cancer.

## 1. Introduction

Gastric cancer is the fourth most common type of cancer worldwide; approximately 989,600 new gastric cancer cases and 738,000 gastric cancer-related deaths were estimated worldwide in 2008 [1]. Surgical resection is currently the treatment of choice for gastric cancer; chemotherapy, radiotherapy, and gene therapy are considered the main adjuvant therapy methods. Efficacy rates of 50% have been reported for chemotherapy drugs, such as cisplatin (DDP), which are widely used in clinical settings [2, 3]. Although chemotherapy is among the primary methods used to treat gastric cancer, the development of multidrug resistance (MDR) commonly leads to a failure of chemotherapy. Therefore, studying the mechanism of MDR and exploring effect MDR-reversing drug are necessary to overcome the bottleneck of cancer chemotherapy.

Recently studies had shown that phosphorylated cofilin-1 (p-cofilin-1) and cofilin-1 played an important role in MDR of cancer. One study had found that p-cofilin-1 was high-expressed in taxol-resistant cells and chemoresistant primary human ovarian cancer tissues [4]. In another study, p-cofilin-1 also showed high-expression levels in vincristine-resistant human osteosarcoma cell line MG63/VCR, which was upregulated by overexpression of LIMK1 [5]. The cofilin-1 was showed higher expression levels in DDP-resistant non-small cell lung cancer (NSCLC) cell ICR-A549 [6]. Cofilin-1 belongs to the actin-binding protein family, the members of which regulate actin depolymerisation. The main functions of cofilin-1 are the decomposition of actin microfilaments and elevation of the rate of actin depolymerisation, both of which influence actin cytoskeletal remodelling. Moreover, cofilin-1 can induce the transformation of filamentous actin (F-actin) to globular-actin (G-actin), activate mitochondrial damage and calcium overloading, and induce the mitochondrial apoptosis pathway. Notably, p-cofilin-1 must be dephosphorylated to participate in actin depolymerisation and translocation into the mitochondria [7, 8].

In recent years, traditional Chinese medicine (TCM) as adjuvant chemotherapy of cancer drugs in China has been widely used in cancer treatment. TCM has the function of strengthening the effect of chemotherapy, reducing the toxic and side effects, reversing the drug resistance of the tumor. Zuo Jin Wan (ZJW), a TCM formula, showed better therapeutic effects in adjuvant treatment of tumors [9–12]. Besides, ZJW has also the effect of reversing drug resistance in gastric cancer and colorectal cancer cell [13–15]. By pharmacodynamics experiments, the effects of ZJW reversing drug resistance in gastric cancer was proved, but its exact mechanism was still unclear.

In the present study, we identified a novel molecular mechanism by which ZJW inhibits DDP-resistance by inducing the mitochondrial translocation of cofilin-1.

## 2. Materials and Methods

### 2.1. Cell Lines and Cultures

BGC823 and SGC7901 human gastric cancer cells were purchased from the Shanghai Cell Collection (Shanghai, China). Cells were cultured in RPMI 1640 medium (Gibco Laboratories, USA) supplemented with 10% (v/v) foetal bovine serum (Gibco Laboratories, USA), 100 *μ*g/mL streptomycin, and 100 U/mL penicillin at 37°C and 5% CO_2_. DDP was purchased from Sigma (St. Louis, MO, USA).

DDP-resistant SGC7901/DDP and BGC823/DDP cells were induced from SGC7901 and BGC823 cells, respectively, using a concentration gradient method to increase the half maximal inhibitory concentration (IC_50_) of DDP. At first, SGC7901 and BGC823 cells were treated with the culture medium containing 0.05 *μ*g/mL DDP for 24 h. Then, the culture medium containing DDP was substituted for the fresh culture medium. When the cell density reached 80%, cells were digested and passage. The cells were treated with 0.05 *μ*g/mL DDP time and again until the cells can be stable passage in such concentration of DDP. Subsequently, the cells were treated with higher concentration of DDP in turn, until the final concentration of DDP reached 1 *μ*g/mL. The cells were cultured in the culture medium containing 1 *μ*g/mL DDP to maintain its drug resistance. Before every experiment, both DDP-resistant gastric cell lines were cultured in drug-free RPMI 1640 medium for 2 weeks.

### 2.2. Preparation of the ZJW Extracts

The ZJW formula composed of two herbs as Rhizoma Coptidis and Fructus evodiae in a 6 : 1 ratio (w : w). Rhizoma coptidis and Fructus evodiae were from TCM pharmacy of Putuo Hospital, Shanghai University of Traditional Chinese Medicine (Shanghai, China). ZJW extracts were prepared as previously described [13–15]. ZJW was extracted through two 1-hour reflux procedures in ethanol (1 : 8, v : v). The extracted mixtures were subsequently filtered, concentrated, and vacuum-dried at 60°C. The preparation of ZJW extracts was standardised and quality controlled according to the guidelines of the Chinese State Food and Drug Administration.

### 2.3. Antibodies

Anti-cofilin-1 (#5175), anti-p-cofilin-1 (#3313), anti-GAPDH (#2118), anti-*β*-actin (8456), anti-cleaved caspase-9 (#7237), anti-cleaved caspase-3 (#9664) anti-cytochrome c (#11940), anti-PARP (#9532), anti-cox IV (#11967), horseradish-peroxidase- (HRP-) conjugated anti-rabbit (7075), and anti-mouse secondary antibodies (7076) were obtained from Cell Signaling Technology, Inc. (Danvers, MA, USA). Anti-slingshot homolog 1 (ab76943), anti-gamma actin (ab194952), and anti-F-actin antibodies (ab205) were obtained from Abcam (Cambridge, MA, USA). An anti-PP1 (E-9) antibody (sc-7482) was obtained from Santa Cruz Biotechnology (Dallas, TX, USA).

### 2.4. Knockdown of the Cofilin-1 Gene

Three siRNAs, specific for the human cofilin-1 gene (Gene ID: 49472823), were synthesised by Biomics Biotechnologies (Nantong, China). The three siRNA and control sequences were as follows: sequence 1: 5′-GAGUGAGGACAAGAAGAACAU-3′; sequence 2: 5′-CGCCACCTTTGTCAAGATGCT-3′; sequence 3: 5′-GAUUUAUGCCAGCUCCAAGGA-3′; control sequence: 5′-UAAGGCUAUGAAGAGAUAC-3′. For knockdown experiments, 5 × 10^5^ cells were plated per well, in a six-well plate. After 24 h, cells were transfected with the above siRNA at the concentration of 50 nM using Lipofectamine 3000 (Life Technologies, Carlsbad, CA, USA) for 48 h.

### 2.5. Quantitative Real-Time PCR Assay

Total cellular RNA was isolated using Plus RNAiso (Takara Bio, Shiga, Japan); subsequently, a PrimeScript RT reagent kit (Takara Bio) was used to synthesise cDNA. Quantitative real-time PCR (Q-RTPCR) to detect cofilin-1 gene expression was performed according to Tang et al. [8]. The following cofilin-1 primer sequences were used: forward, 5′-AAGGCGGTGCTCTTCTGC-3′; reverse, 5′-TTGACAAAGGTGGCGTAG-3′; TaqMan probe, 5′-FAM-CATCCTGGAGGAGGGCAAGGAGAT-TAMRA-3′. The following GAPDH primer sequences were used as controls: forward, 5′-GGTGGTCTCCTCTGACTTCAACA-3′; reverse, 5′-CCAAATTCGTTGTCATACCAGGAAATG-3′; TaqMan probe, 5′-FAM-CGACACCCACTCCTCCACCTTTGACGC-TAMRA-3′. These primers and probes were purchased from Sangon Biotech (Shanghai, China).

### 2.6. Apoptosis Rate Assay

Apoptosis rates were determined using an Annexin V-FITC/propidium iodide (PI) Apoptosis Detection Kit (BD Biosciences, San Jose, CA, USA). Cells were harvested by trypsinisation, washed twice with cold phosphate-buffered serum (PBS), and incubated with Annexin V-FITC, followed by PI. Flow cytometric analysis was performed on a FACSCalibur system (Becton Dickinson, Franklin Lakes, NJ, USA).

### 2.7. Immunofluorescence Assay

HCT116 cells were seeded on cover slips precoated with 0.01% poly-lysine at a density of 5,000 cells per well in a 24-well chamber. Following treatment with ZJW, the cells were treated in the following sequence: 4% paraformaldehyde for 20 min, 0.1% Triton X-100 for 10 min, 5% bovine serum albumin (BSA) for 60 min, and primary antibodies overnight at 4°C. Subsequently, the cells were washed three times using PBS and incubated with an Alexa Fluor 488-conjugated anti-mouse IgG antibody or Alexa Fluor 555-conjugated anti-rabbit IgG antibody (Life Technologies) for 1 h prior to observation with a fluorescence microscope (Leica, Wetzlar, Germany).

### 2.8. Cell Viability Assay

Cells were plated in 10% FBS RPMI 1640 medium at a density of 5,000 cells/well, in a 96-well plate. Cell viability was analysed using a Cell Counting Kit-8 (CCK-8, Dojindo Laboratories, Kumamoto, Japan), according to the manufacturer's protocol. Absorbances of the wells at 450 nm were read using a plate reader (Bio-Rad, Hercules, CA, USA). Each sample was analysed in sextuplicate, and experiments were repeated three times.

### 2.9. Mitochondrial Membrane Potential Assay and Intracellular Calcium Assay

Mitochondrial membrane potential (ΔΨm) was analysed using JC-1 (Beyotime, Haimen, China) in both flow cytometric and cell fluorescence assays. JC-1, a fluorescent probe that detects mitochondrial membrane potential, forms a red fluorescent polymer upon accumulation in the mitochondrial matrix under conditions of high membrane potential; at a lower membrane potential, JC-1 exists as a green fluorescent monomer. Mitochondrial membrane potential was, therefore, measured as the degree of change in the red/green fluorescence ratio. The intracellular calcium concentration was measured using the Fluo-3 AM assay (Beyotime, Haimen, China). In this assay, the intensity of green fluorescence was used to determine the relative concentration of intracellular calcium. The increasing of intracellular calcium concentration was thought to be the results of mitochondrial membrane potential damage and proapoptosis marker. Both flow cytometric and cell fluorescence assays were also used to measure the intracellular calcium concentration.

### 2.10. Analysis of Caspase-3 and Caspase-9 Protease Activity

Caspase-3 protease activity was measured using a Caspase-3 Activity Assay Kit (Beyotime, Haimen, China), according to the manufacturer's instructions. Similarly, Caspase-9 Activity Assay Kit was used to detect caspase-9 protease activity. The kit was used to generate a standard curve from which the caspase-3/ caspase-9 protease activity of each sample was calculated, after which the relative fold changes in activity were calculated using the control value.

### 2.11. Western Blotting and Coimmunoprecipitation (co-IP) Assay

Total cell proteins and cytoplasmic and mitochondrial protein fractions were extracted using the Cell Lysis Buffer for Western, IP, and Cell Mitochondria Isolation Kit, respectively (Beyotime, Haimen, China). Following separation using sodium dodecyl sulphate (SDS) polyacrylamide gel electrophoresis, proteins were transferred onto polyvinylidene fluoride membranes, blocked with 5% BSA, and incubated with primary antibodies and corresponding HRP-conjugated secondary antibodies in sequence. Labelled membranes were visualised using an Enhanced Chemiluminescent Western Blotting Detection System (Millipore, Billerica, MA, USA). GAPDH and COX IV were used as loading controls for total/cytoplasmic and mitochondrial proteins, respectively. A Dynabeads® Coimmunoprecipitation Kit (Pierce Biotechnology, Rockford, IL, USA) was used for the co-IP analysis. Total cell, cytoplasmic, and mitochondrial proteins were incubated in anti-cofilin-1 antibodies and normal mouse IgG at 4°C overnight. Subsequently, co-IP samples were subjected to western blotting as described above, using the appropriate antibodies.

### 2.12. G/F-Actin Ratio Assay

The cellular G/F-actin ratio assay was referenced to that previously described [16, 17]. Briefly, cells were washed in ice-cold PBS and then suspended in lysis buffer (50 mm HEPES, pH 6.4, 1 mm MgCl_2_, 10 mm EDTA, and 1% Triton X-100) for 5 min and centrifuged for 30 min at 18,000 ×g to isolate the supernatant (containing G-actin). The remaining pellet containing F-actin was washed in PBS and resuspended in an equal volume lysis buffer under vigorous agitation. The G-actin protein from the supernatant and the F-actin protein from the pellet were checked by western blot. The ratio of G-actin to F-actin was quantified by the proteins expression level.

### 2.13. Statistical Analysis

Using the SPSS 13.0 software package (SPSS, Inc., Chicago, IL, USA), data were subjected to a single-factor analysis of variance and Student's *t*-test. The results are presented as means (*X*  ± SDs). *P* < 0.05 was considered statistically significant.

## 3. Results

### 3.1. ZJW Increases the Sensitivity of DDP-Resistant Gastric Cancer Cells to DDP

**Figure 1:**
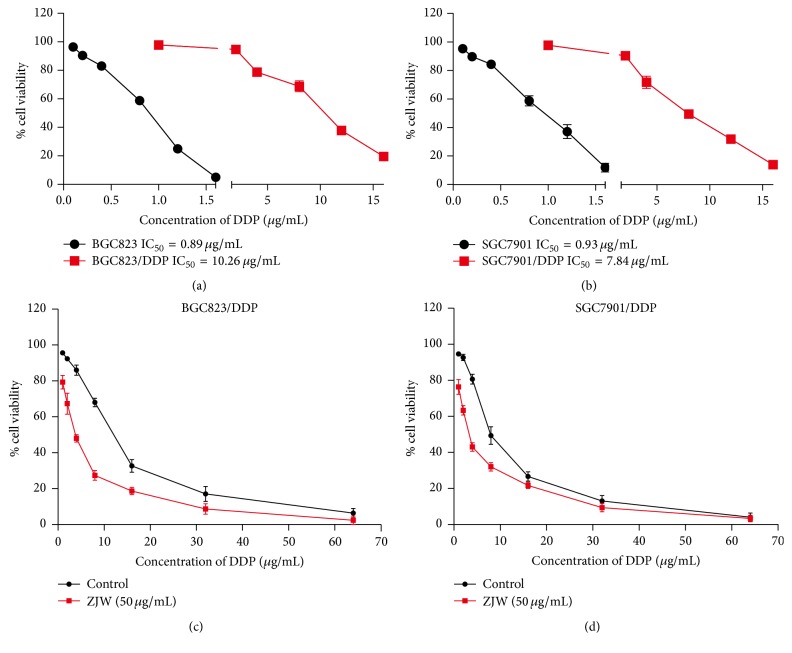
ZJW increases the sensitivity to DDP in BGC823/DDP and SGC7901/DDP cells. (a) The cell viabilities of DDP-resistant BGC823/DDP cells and BGC823 parent cells were measured following exposure to DDP for 48 h. (b) The cell viabilities of DDP-resistant SGC7901/DDP cells and SGC7901 parent cells were measured following the exposure to DDP. (c) and (d) CCK-8 assay was used to detect the cell viability of DDP in BGC823/DDP and SGC7901/DDP cells treated with ZJW (50 *μ*g/mL) and DDP in different concentration for 48 h.

We established the DDP-resistant cell lines BGC823/DDP and SGC7901/DDP by chronic exposure of the DDP-sensitive parent gastric cancer cell lines BGC823 and SGC7901 to low-dose DDP. A CCK8 cell viability assay was then used to detect the inhibitory effects of DDP on BGC823, BGC823/DDP, SGC7901, and SGC7901/DDP gastric cancer cells for 48 h. As shown in Figures 1(a) and 1(b), DDP had significantly lower inhibitory effects on BGC823/DDP (IC_50_ = 10.26 *μ*g/mL) and SGC7901/DDP (IC_50_ = 7.84 *μ*g/mL), relative to their respective DDP-sensitive parent cell lines (BGC823, IC_50_ = 0.89 *μ*g/mL; SGC-7901, IC_50_ = 0.93 *μ*g/mL).

Previous studies have reported that ZJW can reverse the effect of multidrug resistance [13–15]. In the present study, we also observed the ability of ZJW to reverse DDP resistance in BGC823/DDP and SGC7901/DDP cells following treatment with different concentrations of ZJW for 48 h. For BGC823/DDP cells, IC_50_ of DDP decreased from 10.26 to 3.54 *μ*g/mL after ZJW treatment; similarly, IC_50_ of DDP for SGC7901/DDP cells decreased from 7.84 to 2.92 *μ*g/mL. These results indicate that ZJW can enhance the sensitivity of BGC823/DDP and SGC7901/DDP cells to DDP (Figures 1(c) and 1(d)).

### 3.2. Effects of ZJW on Mitochondrial Signalling Pathways

**Figure 2:**
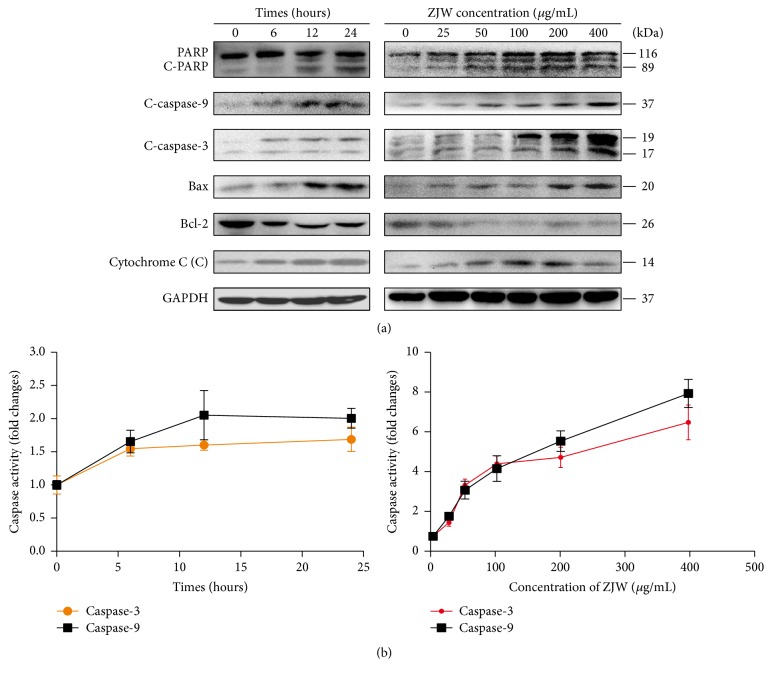
Effects of ZJW on mitochondrial apoptosis signalling pathways. (a) The levels of proteins in mitochondrial apoptosis signalling pathways were evaluated using western blotting in cisplatin (DDP)-resistant SGC7901/DDP cells following treatment with ZJW. Cytochrome C (C) represented the protein level of cytochrome C from cytoplasm. The results indicated dose- (treating time, 24 h) and time-dependent (ZJW, 50 *μ*g/mL) effects. (b) After the same treating method as above, caspase-3 and caspase-9 activity were measured using a standard colorimetric assay (Bio-Rad). Relative caspase-3 and caspase-9 activity levels were normalized against control levels.

Given the role of reversing drug resistance, we hypothesised that ZJW could induce mitochondrial apoptosis in DDP-resistant gastric cancer cells. As the western blotting results in Figure 2(a), ZJW induced caspase-9 and caspase-3 activation and PARP degradation in a time- and dose-dependent manner in SGC7901/DDP cells. These events were accompanied by an increase of the apoptosis promoter BAX, decrease of the apoptosis inhibitor BCL-2, and the release of mitochondrial cytochrome C to the cytoplasm.

We also examined the activities of two key proteases caspase-3 and caspase-9 in the mitochondrial apoptotic signalling pathway by using a Caspase-3 Activity Assay Kit. As shown in Figure 2(b), ZJW induced the activation of caspase-9 and caspase-3 in a time- and dose-dependent manner in SGC7901/DDP cells. These results demonstrate that ZJW can trigger mitochondrial apoptosis in DDP-resistant gastric cancer cells.

### 3.3. ZJW Induces Mitochondrial Injury in SGC7901/DDP Cells

**Figure 3:**
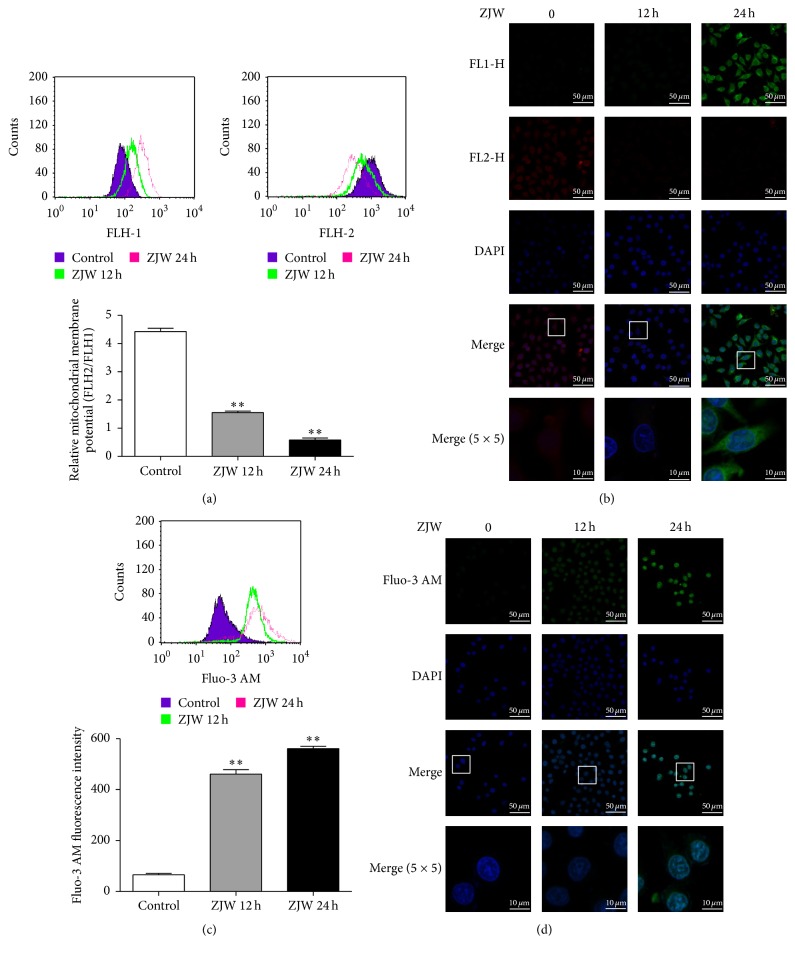
ZJW induces mitochondrial injury in DDP-resistant SGC7901/DDP cells. (a) ZJW decreased the mitochondrial membrane potential (∆Ψm) of SGC7901/DDP cells. SGC7901/DDP cells were treated with 50 *μ*g/mL ZJW for 0, 12, and 24 h. Then ∆Ψm was evaluated using flow cytometry with the fluorescent indicator JC-1 as the degree of change in the ratio of red (FLH-2) to green (FLH-1) fluorescence. ^*∗∗*^ *p* < 0.01, versus control groups treated with ZJW for 0 h. (b) A cell fluorescence assay detected the FLH-2 and FLH-1 fluorescence intensities of JC-1 in SGC7901/DDP cells after exposure to ZJW. (c) ZJW induced intracellular calcium overloading in SGC7901/DDP cells. The intracellular calcium was evaluated using flow cytometry with the fluorescent indicator Fluo-3 AM. ^*∗∗*^ *p* < 0.01, versus control groups treated with ZJW for 0 h. (d) A cell fluorescence assay detected the FLH-1 fluorescence intensity of Fluo-3 AM in SGC7901/DDP exposed to ZJW.

Mitochondrial injury represents an early stage in the mitochondrial apoptosis pathway, characterised by changes in ΔΨm and cytosolic calcium concentration. Accordingly, we detected the ΔΨm and cytosolic calcium concentration of SGC7901/DDP cells using flow cytometry and immunofluorescence, respectively. Using JC-1, we observed an increase in the FLH1 green fluorescence density (JC-1 monomer) and decrease in the FLH2 red fluorescence density (JC-1 polymer), indicating a relative decrease in the ΔΨm (MeanFLH2/MeanFLH1, Figures 3(a) and 3(b)). Using the Fluo-3 AM reagent, we observed an increase in FLH1 green fluorescence density (calcium concentration) (Figures 3(c) and 3(d)). These results suggest that ZJW could induce mitochondrial injury in SGC7901 cells.

### 3.4. ZJW Induces the Dephosphorylation of p-Cofilin-1 in DDP-Resistant Gastric Cancer Cell Lines

**Figure 4:**
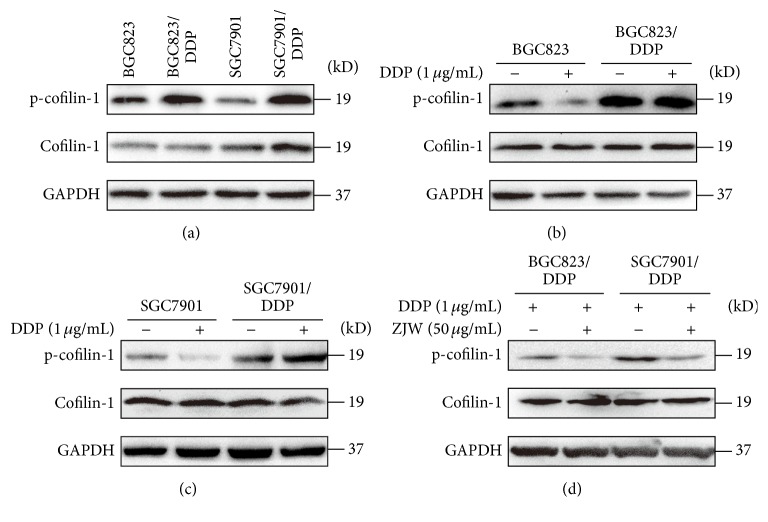
ZJW induces dephosphorylation of p-cofilin-1 in BGC823/DDP and SGC7901/DDP cells. (a) Western blot assayed p-cofilin-1 expressing level in DDP-sensitive cells and DDP resistant cells. (b) and (c) P-cofilin-1 expressing level changes after being treated with 1 *μ*g/mL DDP in four gastric cancer cells for 24 h. (d) The combination of DDP and ZJW induces the dephosphorylation of p-cofilin-1 in BGC823/DDP and SGC7901/DDP cells.

Given the role of p-cofilin-1 and cofilin-1 in drug resistance of cancer [7, 8, 18, 19], we detected p-cofilin-1 and cofilin-1 protein expression levels in the four gastric cancer cell lines. As shown in Figure 4(a), the DDP-resistant cell lines BGC823/DDP and SGC7901/DDP exhibited significantly higher p-cofilin-1 expression levels, relative to their corresponding DDP-sensitive parent cell lines.

After treatment with DDP for 24 h, the expression of p-cofilin-1 decreased significantly in BGC823 and SGC7901 cells, suggesting that DDP could induce p-cofilin-1 dephosphorylation and cofilin-1 activation in DDP-sensitive gastric cancer cell lines (Figures 4(b) and 4(c)). In contrast, the levels of p-cofilin-1 in the DDP-resistant cell lines SGC7901/DDP and BGC823/DDP did not noticeably change, demonstrating that treatment with a lower concentration of DDP could not induce the dephosphorylation of p-cofilin-1. These results suggest that p-cofilin-1 plays an important role in DDP-resistance in gastric cancer cells.

Next, we used western blotting to evaluate the expression of p-cofilin-1 protein in BGC823/DDP and SGC7901/DDP cells treated with DDP and ZJW. Notably, p-cofilin-1 expression significantly decreased after treatment with a combination of DDP and ZJW, although the total cofilin-1 expression level was not significantly changed. These results suggest that the ability of ZJW to modulate DDP resistance correlates with the induced dephosphorylation of p-cofilin-1 (Figure 4(d)).

### 3.5. Translocation of Cofilin-1 and Actin to the Mitochondria Is Induced by ZJW in SGC7901/DDP Cells

**Figure 5:**
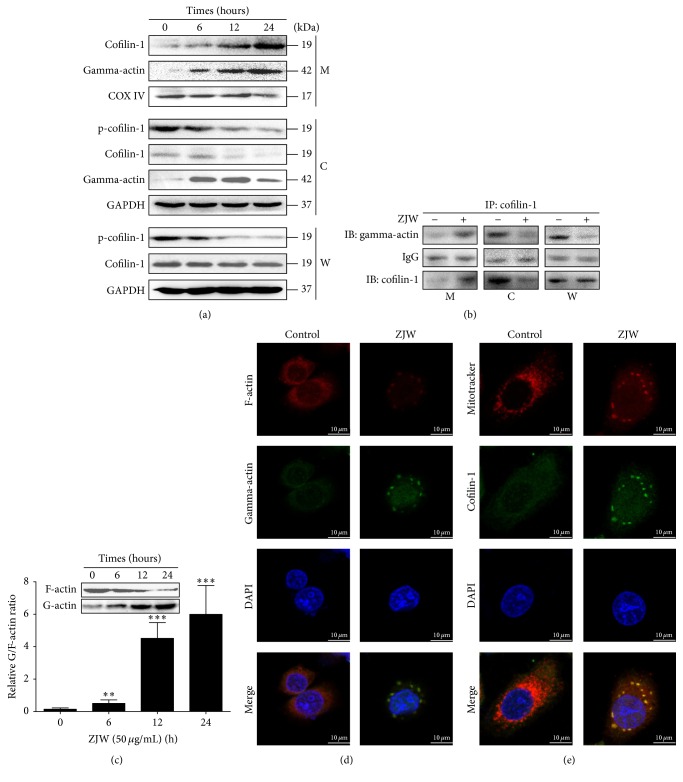
Translocation of cofilin-1 and actin to the mitochondria of SGC7901/DDP cells is induced by ZJW. (a) SGC7901/DDP cells were treated with 50 *μ*g/mL ZJW for 0, 6, 12, and 24 h as indicated. Whole cell lysates and mitochondrial and cytosolic proteins were prepared for the detection of p-cofilin-1, cofilin-1, and gamma-actin using western blotting. The level of each protein was normalised against those of GAPDH (total and cytosolic proteins) or COX IV (mitochondrial proteins). (b) Treated with ZJW (50 *μ*g/mL) for 24 h, a coimmunoprecipitation (co-IP) assay with an anti-cofilin antibody was used to detect the translocation of gamma-actin from the cytoplasm to the mitochondria in SGC7901/DDP cells. (c) Western blot analysis of the G/F-actin ratio changes induced by ZJW in SGC7901/DDP. SGC7901/DDP cells were treated with 50 *μ*g/mL ZJW for 6, 12, and 24 h, respectively, isolating the F-actin extracts and G-actin extracts; the expression of F-actin and G-actin was detected using the anti-F-actin and anti-gamma-actin antibody. ^*∗∗*^ *p* < 0.01 or ^*∗∗∗*^ *p* < 0.001, versus control groups treated ZJW for 0 h. (d) Depolymerisation of F-actin and translocation of G-actin (gamma-actin) from the cytoplasm to the mitochondria was induced by ZJW for 24 h in SGC7901/DDP cells and detected using an immunofluorescence assay. (e) As the same treating method, the ZJW-induced translocation of cofilin-1 from the cytoplasm to the mitochondria (stained with Mitotracker Red) was detected in SGC7901/DDP cells using an immunofluorescence assay.

Previous studies demonstrated that the dephosphorylation of p-cofilin-1 and degradation of F-actin to G-actin, followed by the translocation of actin with cofilin-1 to the mitochondria, were important initiating factors within mitochondrial apoptotic signalling pathways [7, 19]. Accordingly, we first examined the p-cofilin-1, cofilin-1, and actin protein levels in ZJW-treated SGC7901/DDP cells. As shown in Figure 5(a), the expression of p-cofilin-1 decreased after ZJW treatment; in addition, the expression pattern of cofilin-1 shifted from the cytoplasm to the mitochondria. Similarly, the expression pattern of gamma actin (instead of G-actin) also shifted from the cytoplasm to mitochondria. A co-IP assay, with an anti-cofilin-1 antibody, demonstrated an increased amount of actin associated with cofilin-1 in the mitochondria and a concomitant decrease in cytoplasm following ZJW treatment (Figure 5(b)).

Immunofluorescence analysis demonstrated that ZJW induced the degradation of F-actin and the translocation to and aggregation of actin and cofilin-1 in the mitochondria (Figures 5(c) and 5(d)). These findings indicate that ZJW dephosphorylates p-cofilin-1, leading to the degradation of F-actin to G-actin, translocation of actin and cofilin-1 to the mitochondria, and initiation of mitochondrial apoptosis.

### 3.6. Silencing of Cofilin-1 Protects Cells from ZJW-Mediated Apoptosis

**Figure 6:**
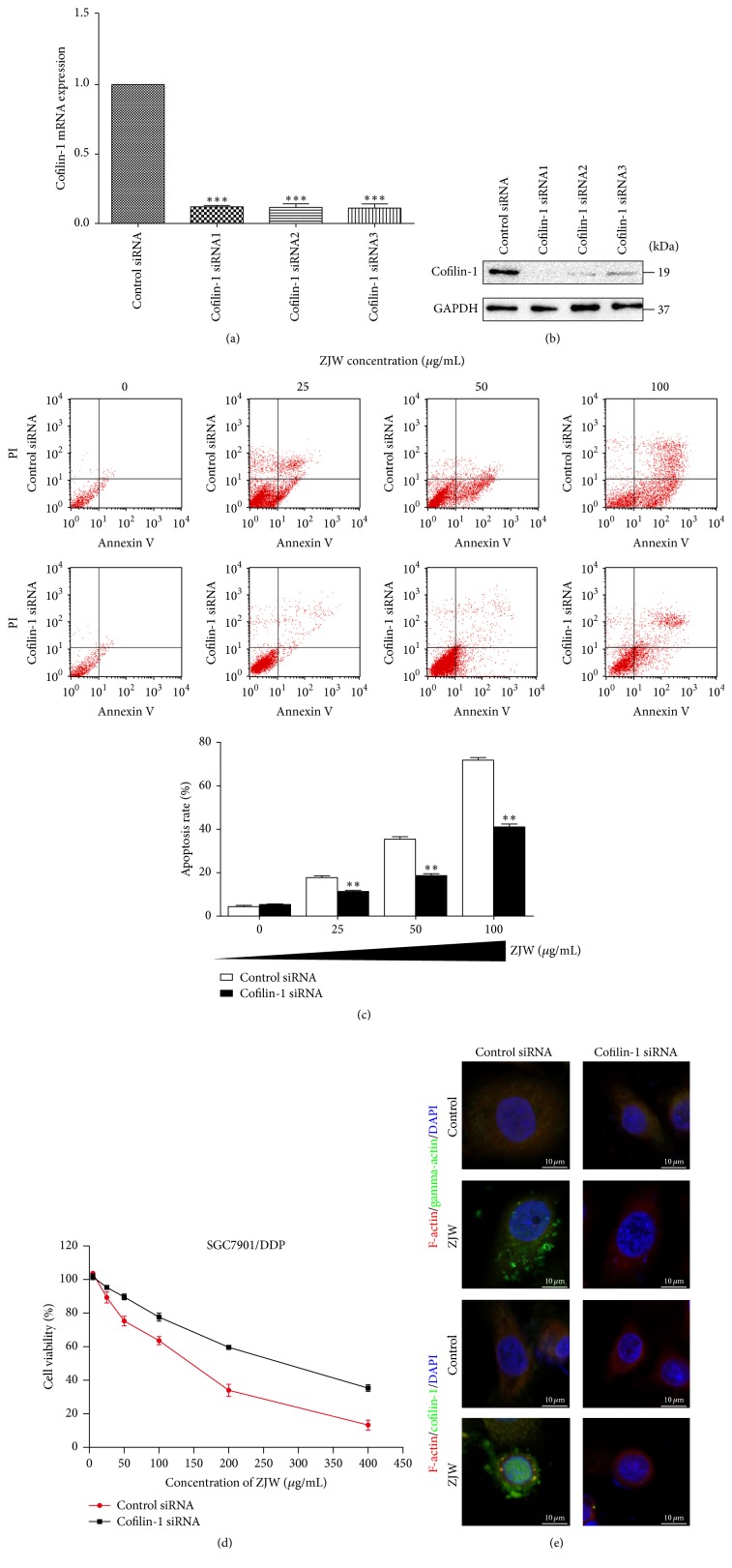
Cofilin-1 knockdown decreases the apoptosis induced by ZJW in SGC7901/DDP cells. (a) The effects of siRNAs specific for the cofilin-1 gene on cisplatin (DDP)-resistant SGC7901/DDP cells were determined using quantitative real-time PCR. ^*∗∗∗*^ *p* < 0.001, versus control groups treated control siRNA. (b) The effects of siRNAs specific for the cofilin-1 gene were determined by western blotting. (c) Flow cytometry demonstrated the dose-dependent effects of ZJW treatment on the apoptosis rate of SGC7901/DDP cells 24 h after siRNA-mediated cofilin-1 knockdown. ^*∗∗*^ *p* < 0.01, versus control groups treated with cofilin-1 siRNA and ZJW (0 *μ*g/mL). (d) The viabilities of SGC7901/DDP cells subjected to cofilin-1 knockdown and exposed to 0, 25, 50, 100, 200, or 400 *μ*g/mL ZJW for 24 h were determined using a CCK-8 assay. (e) SGC7901/DDP cells with cofilin-1 knockdown exposed to 50 *μ*g/mL ZJW for 24 h, depolymerisation of F-actin, and translocation of G-actin and cofilin-1 from the cytoplasm to the mitochondria were detected using immunofluorescence assay.

To verify the role of cofilin-1 in the ability of ZJW to modulate DDP resistance in gastric cancer cell lines, we used RNA interference to reduce the expression of cofilin-1 in SGC7901/DDP cells and subsequently observed the effects of ZJW. We evaluated the expression of cofilin-1 protein and mRNA using western blotting and Q-RTPCR, respectively, at 72 h after transfection with three cofilin-1 siRNA constructs. The results (Figures 6(a) and 6(b)) demonstrated that all three siRNA constructs exerted good inhibitory effects on the expression of cofilin-1 protein and mRNA.

Flow cytometry was used to assess apoptosis in SGC7901/DDP cells treated with different concentrations of ZJW after cofilin-1 knockdown (Figure 6(c)). We observed no significant apoptosis in either cofilin-1 siRNA-treated or control siRNA-treated SGC7901/DDP in the absence of ZJW treatment. ZJW induced apoptosis in both cofilin-1 siRNA- and control siRNA-treated SGC7901/DDP cells in a dose-dependent manner. However, the apoptosis rate was significantly higher in SGC7901/DDP cells treated with control siRNA compared with those treated with cofilin-1 siRNA, indicating the importance of cofilin-1 in the process of ZJW-induced apoptosis.

A CCK8 assay was used to detect the viability of cofilin-1 siRNA-treated SGC7901/DDP cells following treatment with ZJW. As shown in Figure 6(d), ZJW had a significantly stronger inhibitory effect on control siRNA-treated SGC7901/DDP cells (IC_50_ = 134.37 *μ*g/mL) than on cofilin-1 siRNA-treated SGC7901/DDP cells (IC_50_ = 287.43 *μ*g/mL), indicating the importance of cofilin-1 on the ability of ZJW to induce apoptosis and regulate DDP resistance.

Changes in the expression levels and patterns of F-actin, G-actin, and cofilin-1 following ZJW treatment were detected in cofilin-1 siRNA-treated SGC7901/DDP cells using immunofluorescence. As shown in Figure 6(e), in response to ZJW treatment, control siRNA-treated SGC7901/DDP cells exhibited mitochondrial translocation, accumulation of cofilin-1 and G-actin, and lower levels of F-actin, whereas cofilin-1 siRNA-treated SGC7901/DDP cells exhibited a reduced expression of cofilin-1 and G-actin and an increased level of F-actin.

### 3.7. ZJW Induces the Dephosphorylation of p-Cofilin-1 by PP1 and PP2A

**Figure 7:**
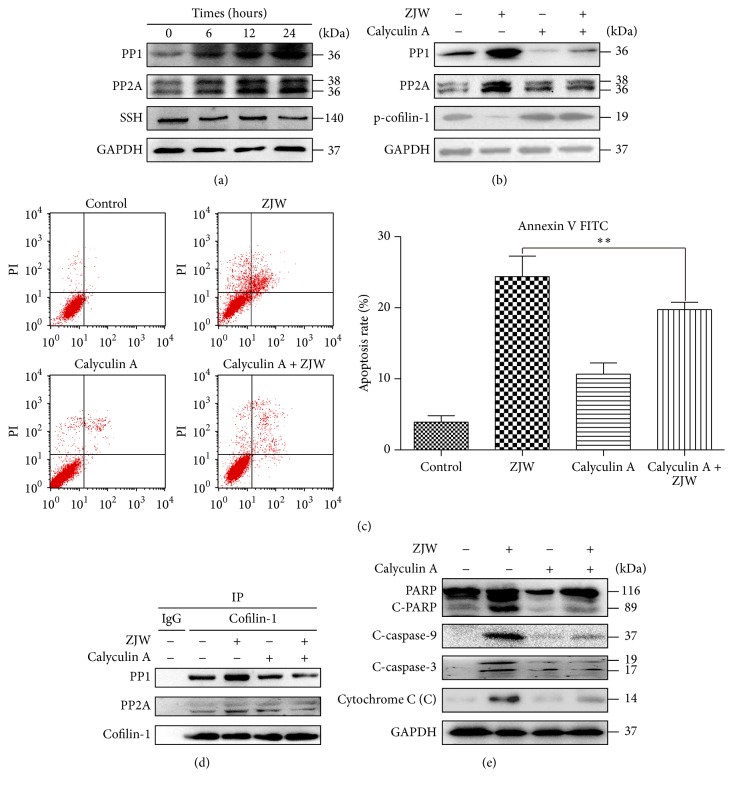
ZJW induces the dephosphorylation of p-cofilin-1 by PP1 and PP2A. (a) SGC7901/DDP cells were treated with ZJW (50 *μ*g/mL) for 0, 6, 12, and 24 h as indicated. Whole cell proteins were prepared and subjected to western blotting to detect PP1, PP2A, and SSH. (b) Cells were treated with ZJW (50 *μ*g/mL) and calyculin A (2 ng/mL) for 24 h. Whole cell protein was subjected to western blotting to detect PP1, PP2A, and p-cofilin-1. (c) Cells were treated with ZJW (50 *μ*g/mL) and calyculin A (2 ng/mL) for 24 h and subjected to an apoptosis evaluation using flow cytometry with an Annexin V-FITC/propidium iodide apoptosis detection kit. ^*∗∗*^ *p* < 0.01, groups treated with ZJW and calyculin A versus groups treated only ZJW. (d) A co-IP assay with an anti-cofilin-1 antibody was used to detect the activation of PP1 and PP2A following treatment with ZJW (50 *μ*g/mL) and calyculin A (2 ng/mL) for 24 h. (e) Western blotting detected PARP, cleaved caspase-3 and caspase-9, and cytochrome C in SGC7901/DDP cells following ZJW (50 *μ*g/mL) and calyculin A (2 ng/mL) treatment for 24 h.

Expressions of the phosphatases PP1 and PP2A and slingshot (SSH), which participate in intracellular phosphorylation reactions, were evaluated in ZJW-treated SGC7901/DDP cells using western blotting. We observed an increased expression of PP1 and PP2A in these cells, whereas the expression of SSH was not significantly altered (Figure 7(a)).

To verify whether the activation of PP2A and PP1 represented a key step in the ZJW-induced dephosphorylation of p-cofilin-1, the PP1 and PP2A inhibitor calyculin A was used to inhibit the activities of these phosphatases. A co-IP assay with an anti-cofilin-1 antibody demonstrated the strong phosphatase inhibitory capacity of calyculin A; specifically, calyculin A treatment reversed the increases in PP1 and PP2A and decreased p-cofilin-1 expression induced by ZJW (Figure 7(b)).

SGC7901/DDP cells treated with ZJW and calyculin A were subjected to apoptosis evaluation. Either ZJW or calyculin A alone could induce apoptosis (Figure 7(c)). However, the combination of ZJW and calyculin A yielded a significantly lower apoptosis rate than that observed with ZJW alone (Figure 7(d)). These results suggested that calyculin A inhibited the apoptosis-inducing effect of ZJW on SGC7901/DDP cells. Finally, proteins associated with mitochondrial apoptosis were detected. As shown in Figure 7(e), calyculin A inhibited the activation of PARP, caspase-3, and caspase-9 and the release of cytochrome C. These results indicate that the activation of PP2A and PP1 plays an important role in ZJW-mediated dephosphorylation of p-cofilin-1.

## 4. Discussion

In this study, we found a significantly higher expression of p-cofilin-1 in DDP-resistant cell lines, wherein a lower concentration of DDP could not induce the dephosphorylation of p-cofilin-1. Moreover, we further determined that ZJW could induce the dephosphorylation of p-cofilin-1 and enhance the DDP sensitivity of BGC823/DDP and SGC7901/DDP cells, inducing apoptosis through mitochondrial signalling pathways, where ZJW promoting the dephosphorylation of p-cofilin-1 in vitro was via the activation of PP1 and PP2A. These results provided further evidence that ZJW could induce apoptosis in DDP-resistant gastric cancer cells.

DDP, a chemotherapy drug commonly used to treat gastric cancer [20–22] acts by inducing mitochondrial apoptosis in cancer cells; however, drug-resistant gastric cancer cells are not sensitive to this agent, and thus, considerable attention has been placed on this issue [23, 24]. Studies have indicated that TCM combination with chemotherapy for cancer can enhance the efficacy of and diminish the side effects and complications caused by chemotherapy. Rhizoma Coptidis is the rhizome of* Coptis chinensis* Franch, which belongs to the Ranunculaceae family being a kind of perennial herb and grows primarily in china. Rhizoma Coptidis contains many kinds of alkaloids, such as berberine, coptisine, methyl coptisine, and palmatine and can cure acute conjunctivitis, acute bacillary dysentery, acute gastroenteritis, hematemesis, furuncle, and other disease. Rhizoma coptidis and its major compounds alkaloid berberine have been reported to reverse drug resistance in cancer [25–27]. Fructus evodiae is of Rutaceae family; its fruit is the herb for ZJW. Evodiamine, an alkaloid extract from Fructus evodiae, has been reported to induce apoptosis and inhibit proliferation in tumors [28–30]. Although the anticancer activity produced by Rhizoma Coptidis and Fructus evodiae and their alkaloids singly used had been verified, the formula ZJW showed much better effect [12]. Therefore, we determined the effect of ZJW on drug resistance in DDP-resistant gastric cancer cells. The results also verified the effect of reversing drug-resistance of ZJW in DDP-resistant gastric cancer cells.

Recent studies demonstrated that high-expression levels of cofilin-1 in many cancers correlated with invasion and metastasis, chemotherapy resistance, and poor prognosis [4, 6, 31–33]. The mammalian cofilin-encoding gene encodes two members of the actin-binding protein family: cofilin-1, which is expressed in various nonmuscle tissues, and cofilin-2, which is mainly expressed in muscle tissue [34]. The main function of cofilin-1 is the decomposition of actin microfilaments; this leads to an increase in actin monomers, which is dependent on the rate of dissociation of the ends of actin microfilaments, and promotes the circulation of actin microfilaments, thus influencing actin cytoskeletal reorganisation and regulating cytoskeletal remodelling. The importance of this process is underscored by the involvement of the actin filament cytoskeleton in many important physiological processes, such as cell growth, differentiation, metastasis, membrane reorganisation, and cell dynamics [35]. Cofilin-1 protein exists in two states, activated (cofilin-1) and inactivated (p-cofilin-1), and these states play different roles in the cell [36]. The dephosphorylation of p-cofilin-1 can induce degradation of F-actin to G-actin and promote the translocation of actin and cofilin-1 complexes to the mitochondria, triggering mitochondrial apoptosis [7, 8]. The phosphoric acid lipases, PP1, PP2A, and SSH, can activate cofilin-1, allowing cofilin-1 to bind F-actin and promote the depolymerisation of actin filaments. Previous studies demonstrated that changes in cofilin-1 or p-cofilin-1 patterns played an important role in multidrug resistance in tumour cells [4, 5, 33]. In this study, high expression of p-cofilin-1 was found in DDP-resistant gastric cells. At the same concentration, DDP could induce p-cofilin-1 dephosphorylation in DDP-sensitive gastric cancer cells but not in DDP-resistant gastric cancer cells. The results suggested p-cofilin-1 as an effective therapeutic target of gastric cancer. Our results thus verified the ability of ZJW to induce the activation of PP1 and PP2A, which further promote the dephosphorylation of p-cofilin-1. Certainly, degradation of F-actin to G-actin, the translocation of actin and cofilin-1 complexes to the mitochondria, and mitochondrial apoptosis were observed in DDP-resistant gastric cancer cells.

## 5. Conclusion

In accordance with our previous studies, we conclude that ZJW can be used as an inhibitor of chemoresistance in gastric cancer, which may partly be due to dephosphorylation of p-cofilin-1 via the activation of PP1 and PP2A. We believe that our study thus demonstrates a naturally derived drug resistance inhibitor in human cancers. In addition, this combination of herbs might yield better results than modern medicine in the context of cancer treatment. ZJW-based treatments should be explored as potential therapeutic strategies for human drug-resistant cancers. Certainly, the cause of high expression of p-cofilin-1 and the mechanism of activation of PP1 and PP2A of ZJW in DDP-resistant gastric cells deserve further investigation.
